# Sexual needs of people with schizophrenia: a descriptive phenomenological study

**DOI:** 10.1186/s12888-023-04640-z

**Published:** 2023-03-09

**Authors:** Jin-wei Yang, Kai Yu, Xiao-qing Wang, Yu Wang, Chen-Chen Zhang, Rui Ma, Hong Yu, Yu-qiu Zhou

**Affiliations:** 1 Department of Nursing, Harbin Medical University, Harbin, Heilongjiang150081 China; 2 Department of Nursing, Fuwai Central China Cardiovascular Hospital, Central China Fuwai Hospital of Zhengzhou University, 450003 Zhengzhou, Henan China

**Keywords:** Sexual needs, Schizophrenia, Psychiatric nursing, Descriptive phenomenology, Qualitative study

## Abstract

**Background:**

Sexual health is one of the main areas of health and basic human rights which has been paid less attention in schizophrenia. Most studies have focused on sexual dysfunction rather than the sexual needs of people with schizophrenia. This study explores the sexual needs of people with schizophrenia and identify factors hindering sexual activities.

**Methods:**

We carried out a qualitative study using a descriptive phenomenological approach. Data were collected at a psychiatric hospital in China. In total, 20 patients with schizophrenia were recruited through purposive sampling. Face to face semi-structured in-depth interviews were conducted with them. Interview recordings were transcribed by the research team, and transcripts were analyzed by two independent coders with Colaizzi’s descriptive analysis framework by using NVivo 11 software. The consolidated criteria for reporting qualitative research checklist was used for reporting.

**Results:**

The data analysis revealed 10 subthemes categorized into 3 macro themes: (1) multiple barriers hinder sexual activity; (2) significance of sex; and (3) conditions for fulfilling sexual needs.

**Conclusion:**

A poor sexual quality of life may be found in patients with schizophrenia. Furthermore, people with schizophrenia did not lose interest in maintaining an active sex life. Mental health services should address this issue in three areas: sexual knowledge, sexual space, and sexual objects.

**Supplementary Information:**

The online version contains supplementary material available at 10.1186/s12888-023-04640-z.

## Background

Schizophrenia is a chronic psychiatric disorder with a lifetime prevalence of approximately 1% [1]. It is often accompanied by cognitive impairment and social dysfunction, such as reduced family role function and a lack of social skills [2]. Negative attributes regarding intimate relationships and sexuality are more in people with schizophrenia than in the corresponding healthy population [3]. Research on the sexuality of people with schizophrenia demonstrated that they have an increased likelihood of experiencing sexual dysfunction( as a symptom of their disorder or side effect of medication) and engage in risky sexual behaviors, such as indulging in sex without using a condom, participating in high-risk sex, having casual sexual encounters, or trading sex for some material gain [4, 5]. It is possible that people with schizophrenia may experience hallucinations or delusions with a sexual content which influence their sexual functioning or behaviour [6]. Moreover, the closed management model of psychiatric hospitals contributes to a lack of privacy, limiting the chance of having a regular sex life [7]. Furthermore, people with schizophrenia often do not have the necessary assertiveness to insist on a safe sexual relationship, which could lead to their sexual exploitation [8].

The World Health Organization defines: “sexual health as a state of physical, emotional, mental, and social well-being in sexuality” [9]. Sexual health requires a positive and respectful approach toward sexuality and sexual relations, as well as pleasant and safe sexual experiences, which are free from coercion, discrimination, and violence. Everyone, regardless of their health condition, must be free to explore their sexuality as they deem fit [10]. Sexual needs be defined as biologically conditioned characteristics of the body, which manifest themselves as desire for sexual satisfaction through relieving recurring psychophysical tension through specific sexual activities that provide the opportunity for experiencing sensual pleasure [11]. Although the ability to perform sexual activities or maintain former sexual life is affected in people with schizophrenia due to their disease or medication, their sexual needs are retained [12]. Studies have shown that 40% of people with schizophrenia need intimate relationships, and 33% continue to need sex [13]. Evidence suggests that unmet sexual needs in people with schizophrenia may hinder their treatment [14], resulting in poor drug compliance and having a major effect on their quality of life [5]. Impaired libido and erectile dysfunction affect the quality of life and self-esteem of these people, which increases the incidence of depression and interpersonal relationship problems [15]. However, mental healthcare providers have poorly addressed the sexual life of these people and have conducted few studies in this regard [16]. Kautz et al. categorized reasons for nurses not discussing peoples’ sexual concerns and problems into four groups, namely inadequate sexual knowledge, attitudes toward sexuality, discomfort in asking about sexuality, and opinions about professional roles and tasks [17].

An accurate estimate of the potential unmet needs of people with schizophrenia is the first step toward creating and implementing policies to address these unmet needs [18]. For mental health professionals, a reliable assessment of sexual needs will aid in clinical decision-making and the development of personalized treatment plans for rational treatment [19]. For patients, it may contribute to promoting adherence to treatment and improving quality of life [20]. For the relationship between healthcare personnel and patients, the assessment of sexual needs will help to provide synergy for interventions, allowing the patients to be understood, respected, and supported [21]. So far, many national and international studies have been conducted regarding different aspects of reproductive and sexual health of people with mental health problems, but no studies have evaluated the sexual needs of people with schizophrenia. In addition, this study further refined the study subjects into people with schizophrenia, who have the characteristics of earlier onset and longer hospital stay when compared with people with other mental disorders, so their sexual needs may be special. Therefore, the present qualitative study was undertaken to explore the sexual needs of people with schizophrenia and their expectations regarding sexual care from the health system. Such information may thus pave the way for further research on sexual needs among people with schizophrenia and try to build further on this theme.

## Methods

### Design

The study followed the phenomenological descriptive method of Colaizzi [22], which is the most suitable method for gaining insight into subjective aspects, that is, understanding the sexual needs of people with schizophrenia by exploring their feelings, experiences, and perceptions [23]. This study was designed and reported and followed the consolidated criteria for reporting qualitative studies (COREQ) checklist (see Table S1).

### Participants

The study was conducted in Harbin, China, from June 2022 to August 2022. Purposive sampling was used to select participants opting for the inpatient and outpatient services of psychiatric hospitals. The inclusion and exclusion criteria for these participants are presented in Table 1. Participants were allowed to choose to be alone or to be accompanied by their families in the interview. In this study, no participant chose to be accompanied by their family.

**Table 1:** Inclusion and exclusion criteria of the participants

Inclusion criteria: 1. Age between 18 and 60 years. 2. Diagnosed by a psychiatrist based on the International Classification of Diseases-10/Diagnostic and Statistical Manual of Mental Disorders-5 criteria. 3. The total score on the Positive and Negative Syndrome Scale changed by < 15, and the total score was < 60 in the past 4 weeks. 4. Provided signed informed consent.
Exclusion criteria: 1. Diagnosed with a mental illness other than schizophrenia, such as depression, dementia, or epilepsy. 2. With debilitating mental disability or illiterate, hampering communication.

### Data collection

The settings were independent, quiet, and private to allow participants to express their experiences freely. A researcher pursuing postgraduation in psychology and mental health who had been trained in qualitative research and interview methods conducted the interviews and collected personal data. The interview outline (Table 2) was initially formulated according to the aim of this study and revised after consulting relevant experts. Moreover, a few steps were taken to improve the interview, which included breaking deadlocks, building trust in relations through verbal and nonverbal words, allowing more time for reflection and response, and ensuring that the participant understood the questions through repetitions and clarifications. The interviews were conducted in the participants’ language (Chinese), and they were interviewed once for 30 to 50 min. Field notes were made during and immediately after each interview. We stopped the interview when the analysis reached data saturation, that is, the interview did not add any new information to the data collection process [24]. Finally, 20 people were included in this study, and no participant refused to participate or quit.

**Table 2:** Outline of the interview

1. What are your thoughts on sex?
2. When was the last time you had sex?
3. Did the disease or treatment affect your sexuality, and if yes, how?
4. How do you want to meet your sexual needs?
5. What difficulties have you encountered in meeting your sexual needs?
6. How would you like your family to contribute to help you fulfill your sexual needs?
7. What would you like the hospital to do for you to help you satisfy your sexual needs?
8. Would you like to say anything about the sexual needs of people with schizophrenia?

### Data analysis

For qualitative studies, data were collected and analyzed simultaneously. We used NVivo 11 to code and manage all interview data. Colaizzi’s phenomenological approach was applied to analyze data with the following steps: (1) reading of interviews by two researchers repeatedly to become familiar with and understand the entire content, (2) identifying significant statements related to sexual needs in people with schizophrenia, (3) extracting meaning fragments through team discussions, (4) organizing significant statements into meaningful units and subthemes into major themes, (5) linking themes closely to research phenomena and detailing them, and (6) providing feedback on the results to participants to ensure the authenticity of the content.

### Trustworthiness

Given the sensitivity of the topic, we established a relationship with the participants by listening to them without judgment, thereby minimizing the influence of the researcher’s beliefs and experiences on the study results. We ensured that the interview questions were neutral and open-ended to allow participants to share their opinions about their experiences related to sexuality. Two trained researchers who spoke the same language as the participants collated and analyzed the data. The study results were discussed with the participants to reach a consensus and were validated by the participants to ensure reliability. For credibility and originality of the study, we recruited participants from across different genders and age groups, with experience of the study phenomenon and with the ability to describe their experiences accurately. The analysis was obtained from interviews, verbatim transcription, and the participants’ own words to ensure that our interpretations of the data were drawn from and evidenced by paradigm extracts from the interview data.

### Ethical considerations

This study was approved by the Ethics Committee of Harbin Medical University (HMUDQ20220517005), and it conformed to the ethical guidelines of the Helsinki Declaration. Before the interview, all the participants received a full explanation of the content and purpose of the study and were ensured confidentiality of the interview content. Written informed consent and recording permission were obtained from all the participants. To respect the wishes and privacy of the participants, they were allowed to decide the length of the interviews, and their names were coded.

## Results

A total of 20 people with schizophrenia were interviewed in this study. The characteristics of the participants are presented in Table 3.

**Table 3:** Characteristics of the participants (N = 20)

Coding	Age (years)	Gender	Marital status	Course (years)	Recruitment sites
A1	27	Female	Divorce	6	Outpatient
A2	28	Female	Single	3	Inpatient
A3	37	Female	Divorce	10	Inpatient
A4	40	Female	Divorce	15	Outpatient
A5	52	Female	Divorce	22	Inpatient
A6	46	Female	Divorce	15	Outpatient
A7	33	Female	Married	11	Inpatient
A8	36	Female	Married	12	Inpatient
A9	38	Female	Married	15	Outpatient
A10	44	Female	Single	22	Inpatient
A11	25	Male	Single	2	Outpatient
A12	23	Male	Single	2	Inpatient
A13	36	Male	Divorce	10	Inpatient
A14	40	Male	Divorce	13	Inpatient
A15	48	Male	Single	18	Outpatient
A16	42	Male	Single	14	Inpatient
A17	30	Male	Single	9	Outpatient
A18	32	Male	Single	12	Inpatient
A19	47	Male	Married	23	Outpatient
A20	34	Male	Single	8	Outpatient

**Fig. 1:**
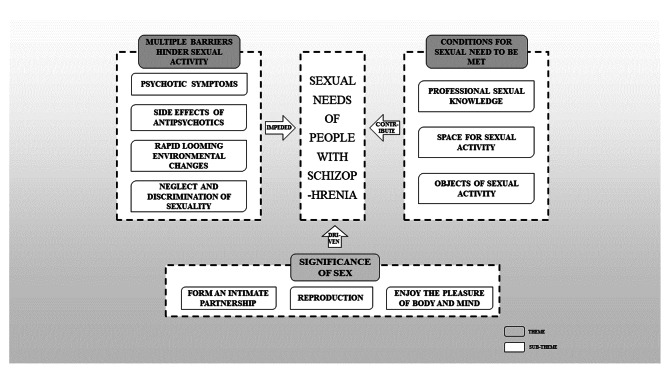
Frame diagram representing the sexual needs of people with schizophrenia

We obtained 10 subthemes that can be fit into 3 themes (Table 4). We found that the sexual needs of people with schizophrenia are driven by their perceptions of sexual activities, including reproduction, formation of an intimate partnership, and enjoyment of physical and mental pleasure. However, they experienced a range of obstacles in sexual life, including psychotic symptoms, side effects of antipsychotics, rapid looming environmental changes, neglect in terms of sexuality, and discrimination. Medical staff can reduce the influence of the aforementioned factors by supporting these people through the provision of conditions to meet their sexual needs (Fig. 1).

**Table 4:** The theme and sub-theme extracted from the data

Theme	Sub-theme
Multiple barriers hinder sexual activity	Psychotic symptoms Side effects of antipsychotics Rapid looming environmental changes Neglect and discrimination of sexuality
Significance of sex	Reproduction Form an intimate partnership Enjoy the pleasure of body and mind
Conditions for fulfilling sexual needs	Professional sexual knowledge Space for sexual activity Objects of sexual activity

### Multiple barriers hinder sexual activity

Multiple factors impede sexual activity in people with schizophrenia. First, psychotic symptoms and side effects of antipsychotics lead to impaired sexual functioning. Second, the closed-space environment prevents people from communicating with the opposite sex and does not provide a private space for sexual activity. Lastly, with the stigma of schizophrenia, the sexual needs of people with schizophrenia are often neglected, and they are discriminated against by their families and even society.

### Psychotic symptoms

Sexuality was severely affected in patients with schizophrenia. When we asked them about their sex lives, one of the main responses was involuntary sexual abstinence, usually linked to a lack of libido:

A7: “Sometimes my husband wants to have sex with me, but I’m just not interested. After a few no’s, he stops asking.”

Schizophrenia can have serious effects on sexuality when there are acute phases such as the inability to recognize one’s partner, having a wrong notion that your partner wants to harm you, or not being aware of what is being done or said:

A12: “I sometimes have an erection in the morning, but I do not care. Because at the moment I am out of control, I’m not too…I’m not very trusting in myself to do anything else.”

### Side effects of antipsychotics

People treated with antipsychotics exhibit various aspects of impaired sexual function, including low libido, erectile dysfunction, and difficulty reaching orgasm. Four participants mentioned that they are unable to ejaculate, experience delayed ejaculation, or have prolonged erection. One participant mentioned a lack of sexual desires:

A19: “When I have sex with my lover, sometimes I cannot get an erection. Even if I have an erection, it does not last for a while. That really sucks.”

A6: “I have not stopped my medication since I got sick. en…I have no libido for a long time, but, I do not know if it’s because of my medication.”

Many participants were aware of the effects of medical therapy on sexual function, but their attitudes about medication were disparate and sometimes conflicting. Some participants decided to stop meeting their own sexual needs to focus on controlling the symptoms of their mental disorder. Conversely, other participants stopped their antipsychotics for sexual pleasure when they had several significant side effects of medication:

A13: “I still have to take medicine to treat diseases. I have prioritized keeping the schizophrenia away from me and wrestling with my work. The main thing is I have to be strong. That’s why I cannot prioritize sex life.”

A14: “Every day is a struggle, and there is no place for sexuality. So I stopped taking pills for sexual pleasure, stopped having sex for a while, and then stopped taking them.”

### Rapid looming environmental changes

Some participants reported that the onset was rather abrupt and resulted in urgent hospital admission and frequent readmissions. The mandatory social isolation was connected to a feeling of being disconnected from the rest of society, preventing them from establishing new social relationships or hindering them from maintaining existing ones:

A15: “When I was in the hospital, I had very little contact with my girlfriend, and then I broke up, and I’ve had very little sexual activity since then.”

A7: “Suddenly locked up in this closed environment, I am not connected to anything personally or spiritually, I am just kind of there.”

Mental health centers have not only strict regulations but also a lack of private spaces where admitted people can develop relationships without feeling constantly monitored. This greatly limited the possibilities of establishing normal social relations. Moreover, if people wanted to have sex, they had no place to do it:

A13: “Once I tried to masturbate in the bathroom, but maybe I had been away from the room for too long, so the nurse found me…So there, there is a real lack of private space.”

### Neglect and discrimination of sexuality

The sexual needs of people with schizophrenia are not only overlooked but also discriminated against by society, which assumes that they have no such needs. Many people suppress their sexual desire and are afraid to express their sexual needs:

A11: “Is it shameful that I want sex? It seems that most people do not understand that people like me also have this need, so even if I have a sexual need, I try to suppress it.”

People with schizophrenia are considered to be emotionally unstable, and certain risks are involved in communicating with them, which further hinders the establishment of their social and intimate relationships, resulting in difficulty in seeking sexual partners:

A20: “They called me a psychopath, I did not have a name, and people thought we were dangerous and would not associate with me, let alone have sex with me.”

## Significance of sex

Under this theme, we identified various motivations for sexual needs in people with schizophrenia. People believed that sex is a prerequisite for reproduction and harmonious or perfect sex life can help build intimate relationships and bring happiness.

### Reproduction

In China, some people consider reproduction as the main purpose of sex. According to the study participants, one of the most important functions of sex is reproduction:

A2: “I think the need for sex is to procreate, and it’s all about having children.”

### Form an intimate partnership

Some of the participants used sex as a medium to express love and as a form of human interaction. In this sense, having sexual relations is contextualized as the expression of a deeply intimate relationship and as a result of being in love with the other person:

A17: “In a relationship, sex is essential if you want to have a deep connection with your partner.”

In addition, sex is the link between two people in a relationship, and the quality of sexual life is crucial in a marital relationship. A harmonious sex life can increase affection which is important for a stable marriage:

A6: “If there is no kissing and no sexual experience between a couple, it is just a shell of a relationship, and sex can make you more loving and harmonious.”

### Enjoy the pleasure of body and mind

Sex is an effective way to regulate emotion effectively and to avoid negative emotions in daily life. The slow, gentle caress between lovers can make them calm down. Some participants said that having a harmonious sex life helps them to temporarily forget their worries and pain:

A8: “I always thought and believed that such intense and happy sexual experiences would make me calmer, I had always had a good sexual relationship with my husband when my sexual function was not so bad, and I feel much happier as a person.”

### Conditions for sexual needs to be met

Under this theme, we mainly explored the hopes and suggestions of people with schizophrenia to satisfy their sexual needs. These included receiving professional knowledge regarding sex, having private spaces where they can enjoy sexual activities, and achieving sexual activities through multiple sexual objects.

### Professional sexual knowledge

Affective and sexual education play a decisive role in people learning to manage their emotions and understand and accept their sexual identity; some participants said that they have no sexual experience. We asked them how they cope with the desires that are constantly arising in them and found that many times they do not even understand what those desires are:

A12: “Sometimes I imagine having sex with people in my head, which makes me restless and irritable, and I do not know how to deal with this situation.”

Although most of the participants strongly emphasized the need for sex-related health education, considering the privacy issues related to sex topics, they lacked the initiative to collect information themselves:

A03: “There is still a lot of doubt about the impact of this disease on sexual life, but I have never talked to others…I mean, it is still too private.”

In addition, medical staff focused on treating the disease and ignored peoples’ sexual needs; sexual education is often scarce or nonexistent in schizophrenia treatment. Some participants stated that if they had received professional knowledge about sexuality much earlier, their lives would have been much different:

A15: “…Male and female, to say we received sexual education—no, not really. Oh no, no doctor or nurse has ever told us this; it was never discussed.”

### Space for sexual activity

Considering the safety, rehabilitation, and medical needs, some people hope that the hospital provides them with a warm and comfortable space for sexual activities:

A09: “How to say…if there are conditions, I hope to be provided a warm and comfortable place so as to help me enjoy the process (sexual activity) and get a pleasant experience.”

### Objects of sexual activity

For married people, their spouse was their primary sexual partner:

A19: “I want to get out of the hospital quickly, go home and have a good life with my wife, and then the sexual needs will be solved.”

Some participants reported that masturbation is one of the ways to deal with sexual needs in the absence of a sexual partner. They hoped that hospitals provide sex toys to help them improve their sexual experience:

A14: “In this hospital, there is no contact with women; masturbation is a way to vent. I will feel good if the hospital provides some sex toys if they are allowed.”

Homosexual behavior was found to be one of the ways of sexual release in male people with schizophrenia. Some participants said that they indulged in homosexual behavior at least once. They sometimes chose to hide part of their sexual identity, which could make them hostile to others:

A18: “In this ward, I had sex with a man I had a good relationship with. At that time, I felt excited and nervous, but…I did not dare to tell others about it. Others would oppose or even discriminate against us; it was just making me too hostile.”

## Discussion

The main finding of this study is that the sexual activity of people with schizophrenia is mediated by several factors, with the main ones being the complications of schizophrenia itself, the side effects of antipsychotics, and the rapid looming environmental changes from home to hospital. People construct their reality based on the interaction of these factors. This is in agreement with the results of studies from European countries [25]. Moreover, our study found that sexuality in people with schizophrenia has been a controversial topic in all quarters of society, which leads to the repression of sexual needs in people with schizophrenia. This has resonance in the Chinese context, particularly where a strong need for sex is generally condemned by society [26], and this view is aggravated by stigma toward schizophrenia [27].

In this study, some participants still considered sex to be a significant basic need of human beings, which constitutes their motivation for sexual needs. On the one hand, sex is an integral part of reproduction, and this belief was more frequent in nulliparous and younger people, who regard healthy sex life and reproduction as extremely important components of a good quality of life and general well-being [28]. On the other hand, participants generally believed that sex plays an important role in enhancing human bonding, which includes love, belonging, and partner communication. These results are in agreement with those of the study by Leeba et al. [29]. Importantly, our study found that a satisfactory sexual life is beneficial for the recovery of people, partly because it can improve psychological statuses such as increasing self-confidence, enjoying family life, and promoting treatment compliance [30].

Sex education is a basic need for all members of society in China, and people with schizophrenia are no exception [31]. However, this need might be more unmet in people with schizophrenia than in the general public [32]. This is partly because people fail to educate themselves and partly because of health provider negligence [33]. Our participants pointed out that being proactive and voluntarily seeking help are uncommon among people with schizophrenia. It can be explained by the Chinese culture, which regards sexuality as a private matter that should not be discussed in public [34]. In addition, the medical staff has not taken the sexual needs of people with schizophrenia seriously. Similar results were found in the Raisi study [35]. Controlling symptoms is more important than addressing sexual problems during the hospitalization of patients and in patients admitted in the acute phase of illness [36]. However, in subsequent session, clinicians must pay attention to sexual problems. This implicitly reflects the problem in psychiatric services. To deal with these problems, an environment where people can freely discuss the topics of sex is essential. In addition, normalizing the inclusion of sensitive topics during treatment after a schizophrenia attack is necessary. Chivilgina et al. argued that digital health intervention in schizophrenia is advantageous [37]. Therefore, we suggest establishing sexuality consultation rooms in psychiatric wards or special websites to provide professional guidance associated with sexuality. Improved collaboration among psychologists, primary care providers,and sex therapists is also needed to best serve the sexual education needs of people with schizophrenia.

In this study, some participants expressed the hope that the hospital would provide them with a space for sexual activity. Studies have suggested that family rooms could be set up in wards, where those who are married or have a sexual partner are allowed to visit for short periods [38]. For single people with sexual needs, a solution is a masturbation which is consistent with the results of the study by Burke [39]. Research shows that after reaching orgasm in masturbation, people’s continuous tense sexual excitement state can be relieved, alleviating their anxiety [40]. Therefore, our study suggests that on the premise of educating people about moderate, safe, and hygienic masturbation, a healthy sexual release system must be established, private space and stimuli (as per people’s preference, such as pictures or videos) must be provided, people must be taught to prepare their own towels or wet wipes, and must be asked to preferably be in a sitting position on the floor or desktop for release. Some participants have same-sex sexual behaviour. Previous research highlight the controversy generated by this type of relationship. They emphasize that although the freedom of people to choose their sexual partners must be respected, protecting people unable to give consent is crucial [41]. In addition, effectively identifying people who have sexual behavior and guiding them to pay attention to sexual hygiene, prevent HIV and other sexually transmitted diseases, and avoid genital injury is important [42].

### Limitations of study

This study has a few limitations. First, for ethical reasons, we included people who were willing to share, discuss, or focus on sexuality. This introduces a selection bias and reduces the external validity of the study. Second, although the sexual need is strongly related to age, the age difference was not adjusted in the studies. Lastly, the interview data must be analyzed from the perspectives of medical staff and spouses or sexual partners of people with schizophrenia.

## Conclusion

This study provides valuable information on the aspects of sexual needs in people with schizophrenia. People with schizophrenia are unable to meet their sexual needs and lack the freedom to choose their partners. Our findings suggest multiple ways by which mental health teams can improve their people’ s sexuality and sexual experiences, including providing comprehensive sexual education and private spaces for sexual activity. It is important that health providers ask and are responsive to the people’ wishes about sexual needs.

## Electronic supplementary material

Below is the link to the electronic supplementary material.

Additional file 1: Table S1 Consolidated criteria for reporting qualitative studies (COREQ): 32-item checklist

## Data Availability

Due to the privacy of the participants involved in the study data, the datasets generated and/or analyzed in the study are not currently publicly available but are available from the corresponding authors of this study upon reasonable request.
