# Supplementary material for: Sexual needs of people with schizophrenia: a descriptive phenomenological study
Source: BMC Psychiatry. 2023 Mar 9;23:147. doi: 10.1186/s12888-023-04640-z (PMC9996993; doi:10.1186/s12888-023-04640-z)
Supplement: Supplementary file 1 — Additional file 1: Table S1 Consolidated criteria for reporting qualitative studies (COREQ): 32-item checklist [file 12888_2023_4640_MOESM1_ESM.docx]

Table S1 Consolidated criteria for reporting qualitative studies (COREQ): 32-item checklist

| Domain 1: Research team and reﬂexivity | |  |
| --- | --- | --- |
| Personal Characteristics | |  |
| 1. Interviewer/facilitator | Which author/s conducted the interview or focus group? | √ |
| 2. Credentials | What were the researcher’s credentials? E.g. PhD, MD | √ |
| 3. Occupation | What was their occupation at the time of the study? | √ |
| 4. Gender | Was the researcher male or female? | √ |
| 5. Experience and training | What experience or training did the researcher have? | √ |
| Relationship with participants | |  |
| 6. Relationship established | Was a relationship established prior to study commencement? | √ |
| 7. Participant knowledge of theinterviewer | What did the participants know about the researcher? e.g. personal goals, reasons for doing theresearch | √ |
| 8. Interviewer characteristics | What characteristics were reported about the interviewer/facilitator? e.g. Bias, assumptions,reasons and interests in the research topic | √ |
| Domain 2: study design | |  |
| Theoretical framework | |  |
| 9. Methodological orientation and Theory | What methodological orientation was stated to underpin the study? e.g. grounded theory,discourse analysis, ethnography, phenomenology, content analysis | √ |
| Participant selection | |  |
| 10. Sampling | How were participants selected? e.g. purposive, convenience, consecutive, snowball | √ |
| 11. Method of approach | How were participants approached? e.g. face-to-face, telephone, mail, email | √ |
| 12. Sample size | How many participants were in the study? | √ |
| 13. Non-participation | How many people refused to participate or dropped out? Reasons? | √ |
| Setting | |  |
| 14. Setting of data collection | Where was the data collected? e.g. home, clinic, workplace | √ |
| 15. Presence of non-participants | Was anyone else present besides the participants and researchers? | √ |
| 16. Description of sample | What are the important characteristics of the sample? e.g. demographic data, date | √ |
| Data collection | |  |
| 17. Interview guide | Were questions, prompts, guides provided by the authors? Was it pilot tested | √ |
| 18. Repeat interviews | Were repeat interviews carried out? If yes, how many? | √ |
| 19. Audio/visual recording | Did the research use audio or visual recording to collect the data? | √ |
| 20. Field notes | Were ﬁeld notes made during and/or after the interview or focus group? | √ |
| 21. Duration | What was the duration of the interviews or focus group? | √ |
| 22. Data saturation | Was data saturation discussed? | √ |
| 23. Transcripts returned | Were transcripts returned to participants for comment and/or correction? | √ |
| Domain 3: analysis and ﬁndingsz | |  |
| Data analysis | |  |
| 24. Number of data coders | How many data coders coded the data? | √ |
| 25. Description of the coding tree | Did authors provide a description of the coding tree? | √ |
| 26. Derivation of themes | Were themes identiﬁed in advance or derived from the data? | √ |
| 27. Software | What software, if applicable, was used to manage the data? | √ |
| 28. Participant checking | Did participants provide feedback on the ﬁndings? | √ |
| Reporting | |  |
| 29. Quotations presented | Were participant quotations presented to illustrate the themes / ﬁndings? Was each quotation identiﬁed? e.g. participant number | √ |
| 30. Data and ﬁndings consistent | Was there consistency between the data presented and the ﬁndings? | √ |
| 31 Clarity of major themes | Were major themes clearly presented in the ﬁndings? | √ |
| 32 Clarity of minor themes | Is there a description of diverse cases or discussion of minor themes? | √ |
